# Analysis of oxidative stress during the menstrual cycle

**DOI:** 10.1186/1477-7827-11-74

**Published:** 2013-08-02

**Authors:** Umberto Cornelli, Gianni Belcaro, Maria Rosaria Cesarone, Annarosa Finco

**Affiliations:** 1 Loyola University School of Medicine-Chicago, Chicago, USA; 2 University of Chieti, Chieti, Italy; 3 Cor Con. International Srl Res Department, Parma, PR, Italy

**Keywords:** Eumenorrheic, Hydroperoxides, d-ROMs test, Estrogens, Progestagens

## Abstract

**Background:**

Few data concerning the oxidative stress (OS) in plasma during the entire menstrual cycle of eumenorrheic women are available.

**Methods:**

OS was assessed in 20 healthy volunteers during the phase of the menstrual cycle by determining the plasmatic hydroperoxides levels (d-ROMs test). The assessment was performed every three days, starting from the first day (t1) up the end of the menstrual phase (t27). Concomitantly, the estrogen (E2) and progestin (P4) levels were determined at the same time intervals.

**Results:**

From a base value (t1) of 284 +/− 38.0 CARR.U., which is essentially within the normal range (<300 Carratelli units or CARR.U.), the OS levels progressively increased to 378 +/− 115 CARR.U. at t15, and then slightly decreased over the subsequent time but with average values >300 CARR.U. Analysis of the E2 levels showed that the maximum OS values were noticed near the estrogen peak, while remaining above the base levels, and then decreased during the progestin phase until returning to normal at the end of the menstrual cycle.

**Conclusions:**

It may concludes that the healthy women go into OS for 2/3 of the menstrual cycle.

## Background

Few data on the variation of oxidative stress across the menstrual cycle in eumenorrheic women have been published [1,2] and no statistically significant differences have been detected on markers such as MDA and linoleic peroxidation derivatives. The markers analyzed were related exclusively to lipids peroxidation, whereas the condition of oxidative stress is affecting many other compounds such as proteins, DNA and sugars. These derivatives taken all together can give a more sensible picture of OS, as it is the case of the hydroperoxides levels in plasma which allow a more complete although nonspecific evaluation of OS [3].

Basing the analysis on the local concentration of GSH (reduced glutathione), GSSG (oxidized glutathione), GSHpx (glutathione peroxidase enzyme) as well as MDA (malonyldialdehyde), Serviddio and colleagues were able to evaluate the change in the oxidative conditions [1] with regard to the menstrual cycle phases. These conditions were then correlated with the estrogen (E_2_) and progestin hormone levels (P4), as well as the luteinizing hormone (LH) and follicle-stimulating hormone (FSH).

This data shows that the peak OS phase occurs in the central phases of the cycle (late follicular phase and early luteal phase), or rather at the time of ovular maturation and possible implantation. This phase occurs with: a) an increase in the production of GSHpx; b) a reduction in the GSH; c) an increase in the GSSG; d) a substantial stability of the MDA.

This all seems to indicate that the increase in OS corresponds to a compensation by the antioxidant systems so that the MDA concentration (index of lipid peroxidation) remains constant; in other words, the oxidation/antioxidant protection system tends to be equilibrated for the entire menstrual cycle. The peak OS will correspond to the E_2_ and LH peaks, while the P4 peak seems to correspond with an OS recovery phase.

The increase in the GSHpx actually corresponds to an increase in the oxidation of GSH; therefore, an increase in the enzyme that “consumes” the GSH to transform the hydrogen peroxide (H_2_O_2_), corresponds to a reduction in the GSH itself, with a resulting increase in the GSSG. This all means OS; it is therefore incorrect to consider GSHpx (any type 1 or 3) as an index of antioxidant capacity, since it is caused by an increased need to use GSH to confront the OS.

When the GSHpx system, SOD (superoxide dismutase) and CAT (catalase) in the erythrocytes are simultaneously analyzed over the course of the menstrual cycle [4], the levels of GSHpx increase in the late follicular phase and initial luteal phase with respect to the other phases, while the SOD and CAT levels remain essentially constant with no significant cycle dependent changes. Analysis of the relationships with the levels of E_2_ indicate that the levels of GSHpx are time related; in extreme synthesis, the increase of one is contemporaneous to the increase of the other. On the contrary, the plasmatic levels of FSH, LH, P4, testosterone and androstenedione do not show any time related relationships with those from the erythrocytic GSHpx and no significant variation in SOD and CAT levels.

From what has been observed, it seems that the isolating action of the E_2_ is not the antioxidant action, but is exactly the opposite [5], which will correspond to the cellular activation needed for the increased energy production from the secretion phase, with a subsequent increase in the production of reactive species such as superoxide (O_2_^•^), H_2_O_2_ and hydroxyl radicals (OH^•^).

If the ovule is not fertilized, the luteal phase is also characterized by an increase in cellular activity and the proliferation of tissue, which precede the end of the cycle, returning the uterus' mucous membrane to the initial cycle condition with the menstrual flow. Locally, the luteal phase occurs with different activations with respect to the estrogen phase, for example, the increase in mucous’ viscosity or in vascular components, and so on. It remains, in any case, similar in terms of oxidation, because it is proliferative phase too, although with different types of final products (mucous, vascular efficiencies). This phase is dominated by the production of P4, which adds to that of the E_2_, and is therefore the highest combined expression of estrogen-progestin steroids.

The plasmatic conditions of OS have never been continuously examined during all phases of the menstrual cycle; it is therefore unknown if that which occurs locally or at the erythrocyte level is also expressed in a similar way in the plasma.

The purpose of this research is to assess the levels of oxidative stress during the normal menstrual cycle. OS condition has been measured by determining the plasmatic hydroperoxides thought d-ROMs test [6,7].

The most common markers for the evaluation of OS are MDA (TBARS), isoprostanes, carbonylated proteins and oxidized DNA. There is no current consensus about which method is the most useful, reliable and specific for the oxidative insult. The suggestion given by some authors is to use more than one test for a more complete evaluation. The choice of hydroperoxides measurement belongs to the consideration that most of the proteins, DNA and lipids once oxidized are transformed into hydroperoxides (ROOH). These compounds are slowly reactive and can transfer the oxidative stress across the cells [8].

A comparative analysis has been done among the most common markers [3] reaching the conclusion that d-ROMs test seems to be the most reliable.

## Methods

20 eumenorrheic female volunteers were enrolled after a written and informed consent. The study received also an institutional review board approval from the ethics committee of the University of Chieti. They were apparently healthy volunteers, most nulliparous (14 of 20), not following any type of contraceptive therapy. The use of any therapy with dietary supplements was considered grounds for exclusion, as was the excessive consumption of alcohol (>3 alcohol units or AU; considering 1 AU =120 mL of wine or 330 mL of beer or 40 mL of spirits), tea (>3 cups/day), coffee (>3 cups/day), chocolate (>50 g/day). The exclusion criteria was also extended to women with a history of surgery, with gynecological diseases with recent infections or with restrictive diet.

### Analysis of oxidative stress (OS)

The analysis of the OS condition during the menstrual cycle was assessed on the basis of the Reactive Oxygen Metabolites-derived compounds (d-ROMs) test [4,5] which is used to determine the levels of hydroperoxides in plasma (expression of lipid peroxidation). The analysis was assessed on patients fasting since the evening before the exam.

The blood was taken by pricking the finger and collecting 0.15-0.2 mL of blood in a heparinized microcuvette. The microcuvette was immediately centrifuged (6000 g for 1 min) in order to isolate the plasma on which the test is performed, within and no longer than 2 h from being drawn. The levels of hydroperoxides were quantified in Carratelli Units (CARR.U.; 1 CARR.U. = 0,08 mg H_2_O_2_/100 mL).

The normal values are <300 CARR.U. The test was applied from the first day from the end of the menstrual flow (t_1_ or start of the new menstrual cycle) and then every three days (indicated as t_1_,t_3_, t_n…_t_27_), for the rest of the cycle, up until the time the next menstrual flow completely stopped. The time progression of the OS during the menstrual cycle can be defined in this manner.

### Analysis of the E_2_ and P4 levels

Only 10 volunteers out of 20 patients accepted to repeat the venous blood sampling and on these patients the protocol also included the evaluation of the E_2_ level at times t_6_ , t_9_, t_12,_ t_15,_ t_18_, t_21_, as well as the levels of P4 at t_12 ,_ t_18_, t_21_, t_24_, in order to determine the correlation of OS with the secretion and luteal phases of the menstrual cycle.

The levels of E_2_ and P4 were determined using commercially available kits (Estradiol: Catalog No KE2D1; Progesterone: TKPG1; Inter Medico Markham, Ontario-Canada). The detection limits for these tests were 5 pg/mL for E_2_ and 0.1 ng/mL for P4, with variation coefficients of 13.1% for E_2_ and 6.5% for P4. The blood for these hormones (2 aliquots of 5 ml each) was taken from the arm vein in heparinized test tubes. All blood withdrawals were done in the morning between 8:00 and 9:30 a.m. with analytical determination within that same day. The volunteers were told to avoid eating large amounts of food the night before the laboratory tests.

### Statistical analysis

From previous experience on d-ROMs test [5] for α = 0.05 and 1-β=0.9 a sample of 12 cases is sufficient to discriminate groups with a power >0.9. The average values and dispersion parameters (SD) were calculated on all data. The comparison between the base values and those at the various observation times were done using Randomized Block Design Anova, the multiple comparisons were calculated using “Dunnett’s Two-Sided Multiple Comparison Test vs t_1_ for d-ROMs test, vs t_6_ for E2 and vs t_12_ for P4. The relationships between the d-ROMs test and E_2_ or P4 levels based on the parametric correlation test.

## Results

Despite the limited number of patients (n = 20), all volunteers finished the study. The general characteristics of the volunteers are reported in Table 1. The OS increase (see Table 2) begins on average between t_6_ and t_24_, and the hydroperoxide levels return to the base values from the start of the new cycle at t_27_.

**Table 1:** General characteristics of the volunteers (Mean ± SD)

	**Mean +/− SD**
*Patients*	20
*Age* (*yrs*)	30.8 +/− 5.66
*Weight* (*Kg*)	63.2 +/− 6.66
*Height* (*m*)	1.67 +/− 0.07
*BMI* (*Kg*/*m*^*2*^)	22.6 +/− 1.58

**Table 2:** **d-ROMs test, E**_**2**_**, P4 values at various times in healthy volunteers (average ± SD)**

	**t1**	**t3**	**t6**	**t9**	**t12**	**t15**	**t18**	**t21**	**t24**	**t27**
***d*-*ROMs ****CARR.U.*	284	273	284	299^a^	336^a^	378^a^	326^a^	315^a^	323^a^	276
	+/−	+/−	+/−	+/−	+/−	+/−	+/−	+/−	+/−	+/−
	38.0	47.0	34.6	31.3	66.8	115.6	66.4	56.3	51.1	44.8
***E***_***2***_*pg*/*mL*			99	222^b^	195^b^	134^b^	157^b^	126		
			+/−	+/−	+/−	+/−	+/−	+/−		
			33.7	34.2	39.9	36.2	36.9	38.4		
***P4****ng*/*mL*					1.7		6.3^c^	13.0^c^	5.9^c^	
					+/−		+/−	+/−	+/−	
					0.54		3.0	6.2	3.8	

^a^Dunnett’s Two-Sided Multiple Comparison Test, p < 0.05 t_n_ Vs t_1_.

^b^Dunnett’s Two-Sided Multiple Comparison Test, p < 0.05 t_n_ Vs t_6_.

^c^Dunnett’s Two-Sided Multiple Comparison Test, p <0.05 t_n_ Vs t_12_.

The concomitance of the maximum increase of hydroperoxides corresponds to a delay from three to six days with respect to the estrogen peak. The rising of the P4 levels instead seems to cause a slight reducing effect on the OS when at its hematic peak (t_21_) but not before.

As can be seen from the average values, the increase in the CARR.U. with respect to those from the start of the cycle reach the condition defined as OS (>300 CARR.U.) from t_12_ to t_24_.

With regard to that noted in the literature [9], the E_2_ levels reach a peak between t_9_ and t_12_ while the P4 levels significantly increase between t_18_ and t_21_. Our evaluations extended to t_24_, which is the moment when the inflection of the P4 levels was observed.

No correlations between the d-ROMs test and E_2_ levels were observed; this seems to indicate that the increase in systemic oxidation is not determined by just E_2_, but also by other factors that could not be identified at our experimental conditions (hypothetically referring to levels of LH, FSH, relaxin, etc.).

## Discussion

Browne et al. [2] end up with the conclusion that no significant variation of the OS markers can be shown during the menstrual cycle. However the data were limited to 9 cases with a large variation of BMI (ranging between 20.9 and 34.7) and also the inter-individual coefficient of variation of the OS markers were very high (from 30 to 56%). This means that the power of the analysis was very limited. In previous trials the coefficient of variation of d-ROMs with almost a similar sampling (12 cases) was < 10%, as a consequence we may suppose that the hydroperoxides measure can be more reliable and early in detecting the condition of OS.

From that observed, a woman is in an OS condition for a good portion of the menstrual cycle, starting near her estrogen peak and hydroperoxides tend to decrease slightly when progestin hormone increases, which however does not allow normal levels to be reached. This fact puts all of the findings made up to now into question in terms of OS in pre-menopausal women.

Therefore, the OS indexes for fertile women should be reviewed in relation to the menstrual period.

If OS relates to a particular moment of the menstrual cycle, its correlations with the various pathologies should be corrected in relation to this "oxidative cyclicity". Obviously, that seen for lipid type hydroperoxides may not apply to the other OS markers, such as isoprostanes or DNA oxidation products (8-OHdG for example) and proteins (carbonylated proteins); nevertheless, at least in part, some of these derivatives also have a hydroperoxide component.

All the hypotheses for the reason for this cyclic algorithm are to be investigated. We know that both the estrogen and the progestin phases have an activation phase; the proliferation phase involves an increase in the production and consumption of energy, which continues into the rather luteal phase of vascular development (vascular space) with a substantial production of nitric oxide (NO^•^).

Nitric oxide, besides having a radical nature, is an important mediator for vasodilatation, the relaxation of the myometrium, anti-aggregation action and angiogenic stimulation which occurs by means of VEGF (vascular endothelial growth factor); this in turn stimulates NO synthase (NOS) [10], indicating that the NO^•^ is trying to maintain its level with the self-induction of NOS.

This synthesis, even though it occurs in all menstrual phases as eNOS (endothelial) and iNOS (induced), increases slightly with progestin stimulus [11]. This entire system must be counterbalanced in a very precise manner.

Locally, there is also an increase in the SOD that coincides with this increase in the synthesis of NO^•^. SOD, carrying out the dismutation of O_2_^•^(superoxide), to form H_2_O_2_ + O_2_, inhibits the NO^•^ + O_2_^•^ reaction that tends to produce the ONOO^-^ ion (peroxynitrite) which, besides having a strong oxidizing strength, removes NO^•^ and limits its availability. Nevertheless, the high level of available NO^•^ can however react with O_2_^•^, since the reaction between the two is by far the fastest in the body (1 × 10^-9^ sec) and can generate OS. It is therefore not surprising that the vectorial result of the antioxidant and oxidant production, action of the progestin phase stabilizes on an OS scale. That which occurred in terms of d-ROMs values was seen in all cases analyzed, therefore the data indicating that OS conditions are present for approximately 2/3 of the menstrual cycle phases is considered “solid”.

The connection between OS and the E_2_ and P4 levels is also confirmed by the previous observations, since we know that the levels of hydroperoxide in women taking birth control pills increase significantly [12,13]. This fact must clearly be confirmed through specific studies, but tends to discredit the hypothesis that exogenous estrogens have an antioxidant action [14,15].

The opposite instead seems to be true. Hypothetically speaking, the action of the estrogens may be similar to that which occurs during exertion [16]. In this condition, the LDLs are oxidized but are also more easily up-taken by the blood receptors, or kept under control and partially "diminished" by the muscular antioxidant systems. Same has been described by other authors [17] that suggest estradiol promote the oxidation of LDL and promotes their clearance from the liver. If however their quantity increases, even at the rate of an inefficient antioxidant system, they are subject to vascular uptake (sub-endothelial) triggering the atherosclerotic inflammatory process. The sub-endothelial will in fact be subject to the action of myeloperoxidase (MPO), which are oxidant-producing enzymes.

This justifies the reduction of the cholesterol and the LDL in premenopausal women, which should not be interpreted as greater efficiency of the LDL blood receptors, but as an increased uptake of LDL thanks to their partially oxidized condition (keep in mind that there is a minimum repairable oxidation limit above which triggers the macrophage reaction).

This oxidative balance, already modified under physiological conditions of normal estrogen/progestin secretion (thus during a normal menstrual phase) can lead to an excess of oxidant production when birth control (BC) therapy is followed. This phenomenon is understandable, since any oral administration of estrogen/progestin generates a hematic peak in both, which is not on a physiological scale and therefore the OS can become permanent.

It belongs to previous observations that anti-proteases in general are more sensible than proteases to oxidation, due to presence of an higher amount of methionine in anti-proteases [18]. Since some proteases such as thrombin are pro-coagulant whereas anti-proteases are anticoagulant (serpins), the tendency of an increase of OS will be a pro-thrombotic status.

A protection mechanism from these thrombotic phenomena, besides the maintaining of the vascular patency of the endometrium and its regular distribution of blood, could originate from the presence of a special peptide hormone in women, relaxin (RLX), which could act as a powerful stimulant for the antioxidant systems [19].

Relaxin is a polypeptide with a MW of 6 KDa and is composed of two peptide chains stabilized both internally and between each other by S-S bridges [19]. It is mainly secreted by the corpus luteum and strategically inhibits alterations in the endometrial flow, to prevent ischemia and reperfusion phenomena, both during the cycle's proliferation phase and then during the eventual pregnancy. The ischemia and reperfusion phenomenon causes OS, both by the production of ROS and by chemotactic stimulation.

The action of the RLX works by over-regulating the iNOS and eNOS, besides the angiogenesis in particular - but not exclusively - in the endometrium; this stimulus occurs in both arterial and venous vessels (thus also in vessels without smooth muscle). RLX is active at nanomolecular concentrations, which therefore can be obtained during the endometrial phases by the ovular implantation and by pregnancy. The angiogenic action is typical of RLX and occurs through the release of VEGF and also bFGF (basic fibroblast growth factor).

An important aspect of the action of RLX is the inhibition of the endothelial adhesiveness of the inflammatory cells (lymphocytes, macrophages) as well as platelet aggregation.

Overall, RLX substantially influences the perfusion and hematic distribution in the endometrium, giving it the ideal flow conditions and performing an anti-inflammatory and anti-thrombotic action and opposes the effects of OS; it could therefore perform a protective action against OS.

## Conclusions

Healthy eumenorrheic women go into OS for 2/3 of the menstrual cycle. Oxidative stress is however a physiological condition for women of child-bearing age. This implies that an over-regulation of the OS defense mechanisms is also present. Therefore, all the OS markers should be studied during the various phases of the cycle and parameterized to this phenomenon.

## Competing interests

The authors declare that they have no competing interests.

## Authors’ contributions

Research was designed by CU; BG and MRC were responsible for the volunteers selection and for the E_2_ and P4 determination. The d-ROMs on plasma samples was carried out by FA at the Cor. Con. International Srl Research Labs, that provided all the analytical reagents also. All authors read and approved the final manuscript.
